# Proactive detection of people in need of mental healthcare: accuracy of the community case detection tool among children, adolescents and families in Sri Lanka

**DOI:** 10.1186/s13034-021-00405-2

**Published:** 2021-10-08

**Authors:** Myrthe van den Broek, Puvaneswary Ponniah, P. Judy Ramesh Jeyakumar, Gabriela V. Koppenol-Gonzalez, John Vijay Sagar Kommu, Brandon A. Kohrt, Mark J. D. Jordans

**Affiliations:** 1 Research and Development, War Child Holland, Amsterdam, The Netherlands; 2 Amsterdam Institute of Social Science Research, University of Amsterdam, Amsterdam, The Netherlands; 3 Research and Development, War Child Holland, Colombo, Sri Lanka; 4 Department of Psychiatry, Base Hospital, Valaichchenai, Sri Lanka; 5 Department of Child and Adolescent Psychiatry, National Institute of Mental Health and Neurosciences (NIMHANS), Bangalore, India; 6 Department of Psychiatry and Behavioral Sciences, George Washington University, Washington, USA

**Keywords:** Proactive detection, Gatekeeper approach, Help-seeking, Children, Adolescents, Mental health, Low-and middle-income countries, Sri Lanka

## Abstract

**Background:**

Most children and adolescents in need of mental healthcare remain untreated even when services are available. This study evaluates the accuracy of a new tool, the Community Case Detection Tool (CCDT). The CCDT uses illustrated vignettes, two questions and a simple decision algorithm to support proactive community-level detection of children, adolescents and families in need of mental healthcare to improve help-seeking.

**Methods:**

Trusted and respected community members in the Eastern Province of Sri Lanka used the CCDT in their daily routine. Children and families detected as potentially in need of mental healthcare based on utilizing the CCDT (*N* = 157, aged 6–18 years) were invited for a clinical interview by a mental health counsellor using the Mini-International Neuropsychiatric Interview for Children and Adolescents (MINI-KID). The CCDT results were compared against the results of the clinical interview. The concurrent validity and performance of the CCDT were also evaluated by comparing the CCDT outcomes against the Strengths and Difficulties Questionnaire (SDQ).

**Results:**

7 out of 10 children and families detected by community members using the CCDT were confirmed to be in need for treatment (positive predictive value [PPV] = 0.69; 0.75 when compared to the SDQ). Detections based on the family problem vignette were most accurate (PPV = 0.76), followed by the internalising problem vignette (PPV = 0.71) and the externalising problem vignette (PPV = 0.62).

**Conclusions:**

The CCDT is a promising low-cost solution to overcome under-detection of children and families in need of mental healthcare. Future research should focus on evaluating the effectiveness, as well as additional strategies to improve help-seeking.

**Supplementary Information:**

The online version contains supplementary material available at 10.1186/s13034-021-00405-2.

## Background

Globally, up to 20% of all children and adolescents experience a mental health condition, yet the majority remain untreated [1, 2]. Although the *treatment gap* between the burden of mental health conditions and engagement with appropriate care is universally large [3], it is particularly true for children and adolescents growing up in conflict affected low- and middle income countries (LMICs) [4]. Prevalence rates of psychiatric disorders are estimated to be two to three times higher among conflict-affected populations compared to those in the general population [5]. These elevated levels of psychiatric disorders have not only been attributed to the exposure to traumatic events. Post-conflict daily stressors such as increased poverty rates, loss of family members and caregiver mental health have also been associated with increased levels of psychiatric disorders in children [6]. Furthermore, traumatic experiences from one generation may be transmitted to the next [7].

In Sri Lanka, the context of this study, the adverse impact of the civil war on the mental health of children and adults, family structures and community dynamics has been well documented [8, 9]. A study reported that nearly one in five children aged 13–18 years had experienced a mental health condition [10]. Another study of adults five years after the conflict showed a steady increase in symptoms of depression and anxiety depending on the level of exposure to past conflict [11]. Despite improvements in available decentralised mental health services in Sri Lanka [12], there is a major mental health treatment gap [13]. The most common barriers to seeking help for mental health problems include: a lack of awareness about symptoms or available services; myths about mental health; widespread social stigma; and negative beliefs about help-seeking [12]. It can be especially challenging among children and adolescents, who often rely on others to access care, to distinguish between symptomatic and normal behaviour [14].

Time and financial resources associated with existing methods to detect mental health problems are major deterrents to the feasibility of implementation in most LMICs [15]. Systematic universal screening, for example, often requires assessing all children in a classroom, community or primary health centre. Furthermore, it may exclude most vulnerable children who are out of school or who do not visit a health centre regularly. A proposed alternative method to overcome these demand side barriers is proactive community-level case detection by trusted and respected community members [15–17]. This approach entails the proactive process of identifying, or locating, children in need of mental healthcare from the larger population for the purpose of help-seeking promotion. Instead of screening the entire community, proactive detection relies on informal observations from trusted community members.

The Community Informant Detection Tool (CIDT) was developed to support proactive detection of mental health problems [15]. It uses paragraph-long illustrated vignettes of the most common manifestations of mental health conditions, using culturally acceptable and non-stigmatising language. Trusted and respected lay community members are trained to use the tool in their daily routine. This allows them to proactively detect people in need of mental healthcare and to encourage help-seeking. The evaluation of the effectiveness of this approach among adults in Nepal demonstrated 46.9% greater help-seeking for mental health problems in areas randomized to using the CIDT compared to the control arm [18].

Building on these positive findings among adults, a child-focused Community Case Detection Tool (CCDT) was developed and evaluated in schools in Palestine. Using the CCDT, teachers accurately detected children in need of mental healthcare in three out of four cases (positive predictive value [PPV] = 0.77) [19]. Drawing on these promising findings, we applied the same tool and procedures to community settings as part of the current study. While teachers are likely to have more relevant training and more frequent interactions with children, trusted and respected community members are likely to have more informal encounters and closer relationships with families. Since all gatekeepers are selected based on the same criteria of having frequent interactions with children, and for being a trusted and respected member in the community, we hypothesized a comparable PPV in community settings. Furthermore, given the vital impact of family functioning on the mental health of children [9], an additional vignette was developed and evaluated that focused on family-level problems. Our hypothesis was that the CCDT could also be used to proactively detect families in need of mental healthcare.

## Methods

### Setting

Sri Lanka is a lower-middle income country in South Asia with a multi-ethnic and multi-religious population of 21.3 million. Thirty nine per cent of the population is under the age of 24 and 80% resides in rural areas [20, 21]. The Tsunami in 2004 and three-decades of civil war which ended in 2009, resulted in over 100,000 lives lost and left 300,000 civilians internally displaced [11, 21]. This study was carried out in three divisions in the Eastern Province, with a total population of 25,591 children aged 5–19 years [22]. Despite Sri Lanka’s overall economic growth, poverty rates in the Eastern and Northern Province, where most of the armed conflict was concentrated, are far above the national average [21]. There is no available data on the prevalence of mental health problems among children and adolescents in the Eastern Province. A 2011 study conducted in the Northern and Eastern Province showed that 92% of the children experienced life-threatening events such as bombings, attacks on homes and loss of family members during the conflict [23]. Furthermore, a qualitative study in the Eastern Province reported that adolescents perceived disrupted family relationships, separation and migration of parents, violence at home and sexual abuse as the main factors affecting their mental and physical well-being [24].

### Design

This study assessed the accuracy of the CCDT. The purpose of the CCDT is to support proactive community-level detection of children and adolescents aged 6–18 years and families in need of mental healthcare to encourage help-seeking. This study therefore focused on the accuracy of CCDT probable positive cases (i.e., those detected by community members using the CCDT as probably in need of mental healthcare). A small proportion of CCDT probable negative cases (i.e., those detected by community members using the CCDT as probably *not* in need of mental healthcare) were included to avoid confirmation bias. In addition, concurrent validity of the CCDT positives was assessed against a widely used alternative instrument to detect mental health problems among children and adolescents.

### Instruments

#### Community Case Detection Tool

The CCDT is a tool for trusted and respected community members, who do not have any professional mental health background (‘community gatekeepers’). It uses an adapted version of the ‘prototype-matching approach’ which is originally developed to simplify and standardize diagnosis. Following this approach, the tool presents three context-sensitive prototypes (i.e., case vignettes) of 150–200 words each (see Additional file 1). Each vignette presents a coherent pattern of child mental health problems or family-related problems. The vignettes are paired with six illustrations to support recognition in daily life [25]. At the bottom of the tool a simple decision tree algorithm is presented to determine the follow-up action based on the severity and functional impact of the symptoms identified. The tool is meant to be used as reference material onto which trained community gatekeepers can match children and families they encounter in their daily routine. If there is a match with one of the vignettes, and the symptoms are thought to be impacting daily functioning, the gatekeeper is advised to support the child and family to seek help from available services. In this study, we evaluated three Tamil vignettes focusing on internalising problems, externalising problems and family-related problems. A positive match with one of the three vignettes was scored as ‘CCDT probable positive’.

#### Ten Question Screen for Childhood Disability

An abbreviated four-item version of the Ten Questions Screen for Childhood Disability (TQS) was used to assess hearing, speaking, or severe cognitive disabilities prior to participation in the study. As a screener tool, the ten item version previously showed overall acceptable psychometrics in Bangladesh, Jamaica and Pakistan (sensitivity from 0.53 to 0.84, specificity from 0.85 to 0.92) [26]. The research methods were insufficiently adapted and the research team was not equipped to administer the clinical interview to these children. Data from children who scored positive on one of the four items were therefore excluded from the sample, but were offered the same services if needed.

#### Mini-International Neuropsychiatric Interview for Children and Adolescents (MINI-KID)

The Indian Tamil MINI-KID 6.0 was used to evaluate the mental health of children and adolescents the gatekeepers had detected. The MINI-KID is a short structured clinical interview to assess the presence of current DSM-IV (Diagnostic and Statistical Manual of Mental Disorders) and ICD-10 (International Classification of Diseases) disorders in children and adolescents aged 6–17 years. In previous studies, the test retest and interrater reliabilities have been shown to be good (0.64 to 1), sensitivity ranged from 0.61 to 1 and specificity from 0.73 to 1 for the individual disorders [27]. Each diagnostic module starts with a screener followed by more detailed symptom, severity and functionality questions. The MINI-KID has been used with children in Sri Lanka before [28]. Relevant modules were selected by a child psychologist (MJ), supervising psychiatrist from Sri Lanka (PJRJ) and Indian child and adolescent psychiatrist and master trainer (JVSK). The selected modules were depression, suicidality, dysthymia, panic disorder, separation anxiety disorder, obsessive compulsive disorder, post-traumatic stress disorder, alcohol and substance dependence, attention deficit hyperactivity disorder, conduct disorder, oppositional defiant disorder, generalized anxiety disorder and adjustment disorder. The module on suicide was only administered for children aged 10 years and older and the modules on alcohol and substance dependence only for children aged 13 years and older. These modules were deemed culturally inappropriate for younger children based on feedback from the senior counsellors during the training. Standard relevant scoring and instructions were used for functional impairment caused by the symptoms and the time frame (i.e., current, past 6 or 12 months).

#### Family functioning

We used an adapted version of the Safe Environment for Every Kid—Parent Questionnaire-R (SEEK PQ-R) to assess family problems and child protection needs [29]. Relevant items of the SEEK PQ-R were selected based on the construct captured in the family vignette. The questionnaire was further adapted and translated through a systematic process in which the items were first translated into Tamil. The research team provided feedback to ensure separate items and translations were culturally appropriate, followed by a blind back-translation. The final questionnaire consisted of 14 items that addressed harsh punishment, child neglect, parental stress, intimate partner violence and substance abuse.

#### Indication for treatment

At the end of the interview, the senior counsellor administering the MINI-KID and SEEK PQ-R answered a concluding dichotomous question regarding the need for any psychological treatment from a mental health counsellor or psychiatrist or child protection service. The indication for treatment was scored (i.e., yes/no) based on the counsellors’ judgement following the information provided in the structured clinical interview and the family assessment.

#### The Strengths and Difficulties Questionnaire

The Sri Lankan Tamil parent version of the Strengths and Difficulties Questionnaire (SDQ) was used to assess the concurrent validity of the CCDT positives. This widely used 25-item behavioural screening questionnaire for 3–16 year old children, covers emotional symptoms, conduct problems, hyperactivity/inattention, peer relationships problems and prosocial behaviour. In previous studies, the parent version has shown interrater reliabilities between 0.37 and 0.62 for the different subscales. The Tamil self-report version showed acceptable internal consistency of the subscales (Cronbach’s alphas between 0.67 and 0.78), sensitivity of 0.69 and specificity of 0.92 [30, 31]. A three point Likert scale allows the respondent to indicate how each item applies to the participating child [30]. All items, except those related to prosocial behaviour, generate a total difficulty score classified as SDQ ‘normal’ (i.e., a score between 0 and 13) or SDQ ‘borderline’ and ‘abnormal’ (i.e., a score between 14 and 40).

### Training and supervision

This study was carried out through an existing partnership between War Child Holland (WCH), an international non-governmental organisation (NGO) and the Eastern Self-reliant Community Awakening Organisation (ESCO), a local NGO. Community gatekeepers with regular interaction with children and families participated in a two-day training by the research coordinator (RC; PP). The training covered a basic introduction to child and adolescent mental health, the use of the CCDT and ethical considerations related to proactive case detection such as confidentiality, stigma and child safeguarding.

A master trainer and child and adolescent psychiatrist (JVSK) with extensive experience in conducting the MINI-KID trained a supervising psychiatrist (PJRJ) and back-up psychiatrist for three days. The supervising psychiatrist subsequently trained five senior community counsellors (four female, and one male) for five days to administer the MINI-KID and the SEEK PQ-R. Supervision meetings were held with the counsellors for quality control and to support with referrals. Ten research assistants (RA) were trained for six days in research basics, ethics, informed consent and assent procedures, the SDQ, and data management. All research team members were trained in an adverse events reporting mechanism and the supervising psychiatrist followed up on children and families in need of immediate assistance.

### Participants and procedures

Community gatekeepers in this study were all female and older than 18 years. They included youth club leaders (*n* = 11), women society group members (*n* = 22) and community health volunteers (*n* = 12). Youth club leaders organise recreational and awareness-raising activities in their village. Women society group members mobilise women to improve their social and economic conditions. Health volunteers assist midwives and medical health officers to organise monthly health clinics and conduct home visits. They used the CCDT for six months during their daily routine activities and detected a total of 238 children aged 6–18 years.

After obtaining informed consent and assent, a study ID was created, the TQS was administered and an appointment with the counsellor was arranged by the RA. Within two weeks of identification, the counsellor met with the family at their home or another convenient location to conduct the clinical interview and the family assessment. Children aged 13–18 years were interviewed individually and younger children in the presence of their caregiver. The RA followed up within two days after the counsellors’ visit to administer the SDQ with the same caregiver. Direct contact between the counsellors and gatekeepers was limited to reduce potential confirmation bias. In addition, gatekeepers were asked to identify a small proportion CCDT probable negatives throughout the study period. Counsellors were informed that both positive and negative cases would be referred to them, but not how many or who they were. The CCDT probable negatives were invited to participate in the study following similar procedures, but were only included to minimize confirmation bias.

For the purpose of this study, a referral tracking sheet was developed for community gatekeepers. They were asked to note down the vignette that was used for the identification and their knowledge about the need of mental healthcare for the detected case prior to using the CCDT. Children and families that were known to the gatekeeper as needing mental healthcare prior to the introduction of the CCDT were excluded from the analyses.

### Ethics

Ethical approval was obtained through the Ethics Review Committee of the Faculty of Health-Care Sciences at the Eastern University in Batticaloa. Divisional and district level approval was obtained before the start of this study. Prior to official informed consent and assent procedures, the gatekeepers asked the caregiver whether they were interested in participating in a research study. The referral tracking sheet was only completed for those families that were willing to participate. All children and families were informed about available and free of charge support, regardless of their participation in the study. Help-seeking was only encouraged, never imposed.

### Analysis

The results of the CCDT, MINI-KID, SEEK PQ-R and SDQ were analysed using the Statistical Package for Social Sciences (SPSS version 19.0). The interrater reliability (IRR) of the MINI-KID among the five counsellors was assessed using Krippendorff’s alpha for dichotomous variables [32]. The IRR was calculated for a selection of the screener, diagnostic, indication for treatment items, and total of these items.

The accuracy of the CCDT was assessed through the Positive Predictive Value (PPV), which is calculated as the percent of children and families detected using the CCDT (i.e., probable positives) who *are* in need of mental healthcare based on the clinical interview. The primary reference criterion was the indication for treatment. The secondary reference criterion was a diagnosis of a psychiatric disorder.

The primary outcome of this study was the PPV for all CCDT positives, regardless of the vignette used, assessed against the indication for treatment. The secondary outcome was the PPV for the subsample of CCDT internalising or externalising positives against diagnostic criteria. CCDT positives detected using the family vignette or cases detected with multiple vignettes were excluded from this subsample because diagnosis of a mental disorder is not applicable to these cases. Exploratory analyses were done to assess the differences in PPV for each individual vignette (i.e., internalising, externalising and family vignette), for each gatekeeper group separately (i.e., youth club leaders, women society group members and community health volunteers), for different age groups and gender against the indication for treatment. CCDT internalising positive cases were also compared against selected MINI-KID modules representing anxiety, depressive and somatic symptoms and the CCDT externalising positive cases with modules related to impulsive, disruptive conduct, and substance use symptoms. For the small proportion of CCDT probable negatives, we also assessed the Negative Predictive Value (NPV). This was calculated as the proportion of CCDT probable negative cases that were *not* in need of mental healthcare, against both reference criteria.

Since our study focused on CCDT positives, we could not establish the concurrent validity with a correlation coefficient. It was therefore assessed as the proportion of agreement between the CCDT positives and the SDQ positives, i.e., borderline and abnormal scores. As with other studies in Sri Lanka, we used the internationally applicable original three-band cut-off scores for the SDQ [31]. Additionally, the PPVs of the CCDT were compared to the PPV of the SDQ against the indication for treatment criterion.

## Results

**Fig. 1:**
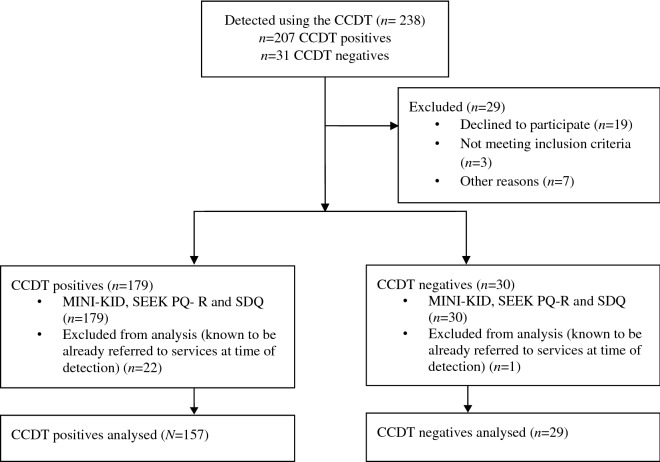
Participant flowchart

A total of 207 CCDT positive children were detected, of whom 27 were excluded because of not providing consent, meeting exclusion criteria based on the TQS or were lost to follow up. Another 23 were excluded from the analysis because the gatekeeper knew about the need for mental healthcare prior to the introduction of the CCDT, which may have influenced the detection (see Fig. 1). Our final sample therefore consisted of *N* = 157 CCDT positives. In addition, 31 CCDT negative cases were detected to avoid confirmation bias by the counsellors, of which two were excluded because of consent.

The average age of our sample was 12.3 years (*SD* = 3.3), with an equal distribution of girls and boys. The specification and frequency of vignettes used and gender distribution are presented in Table 1.

**Table 1:** Frequencies CCDT positives for Each Vignette Used

Vignette used	Total sample	Girls *n* (%)	Boys *n* (%)
Internalizing problem	58	31 (53.4)	27 (46.6)
Externalizing problem	34	9 (26.5)	25 (73.5)
Family functioning problem	53	32 (60.4)	21 (39.6)
Combination: internalizing and family	8	6 (75.0)	2 (25.0)
Combination: all three vignettes	1	0 (0)	1 (100)
Vignette not specified	3	1 (33.3)	2 (66.7)
Total CCDT positives	157	79 (50.3)	78 (49.7)

The IRR using Krippendorff’s alpha was *α* = 0.88 (95% CI; 0.82–0.92) for the total of the selected screener, diagnostic, and indication for treatment items (32 items). For the 12 screener items *α* = 0.75 (95% CI; 0.62–0.86), for the 13 diagnostic box items, *α* = 0.94 (95% CI; 0.88–0.98), and for the seven treatment items *α* = 1.

Of the 157 CCDT probable positives, 109 were indicated for mental health treatment (PPV = 0.694). Analysis against the secondary criterion of psychiatric diagnosis showed that 42 of the 92 CCDT internalising or externalising positive cases were diagnosed with a psychiatric disorder (PPV = 0.457). Exploratory analyses with separate subsamples against the primary criterion showed that detections based on the family vignette returned the least false positives (PPV = 0.755), followed by the internalising problem vignette (PPV = 0.707) and the externalising problem vignette (PPV = 0.618). Further assessment of the PPV against specific diagnostic criteria showed that 21 of the 58 CCDT internalising positives met the diagnostic criteria of any of the relevant modules related to internalizing disorders (PPV = 0.362) and 7 of the 34 CCDT externalising positives (PPV = 0.206) met the diagnostic criteria of any of the externalizing disorder modules.

The measures of concurrent validity showed that 46.7% of the 92 CCDT internalising or externalising positives returned ‘borderline’ or ‘abnormal’ SDQ total difficulty scores. When compared against the same reference standard, there is little difference between the PPVs of the SDQ and the CCDT: of the 67 SDQ ‘borderline’ or ‘abnormal’ cases, 50 were indicated for mental health treatment (PPV = 0.746 vs. PPV = 0.674 with the CCDT). Exploratory PPV analyses for each gatekeeper group and vignette separately and by gender are summarized in Table 2. Of the 29 CCDT negative cases, 21 did not require mental healthcare (NPV = 0.724) and 24 did not meet any diagnostic criteria (NPV = 0.828).

**Table 2:** Positive Predictive Values

Positive Predictive Value		
	Indication for treatment	
	*n/N*	PPV
All CCDT positives	109/157	0.694
Sub samples	*n/N*	PPV
Vignette(s) used		
Internalizing or Externalizing	62/92	0.674
Family functioning	40/53	0.755
Community Gatekeeper		
Youth club leader	22/29	0.759
Community health volunteer	59/93	0.634
Women group members	28/35	0.80
Total CCDT positives	109/157	–
Gender		
Girls	55/79	0.696
Boys	54/78	0.692
Total CCDT positives	109/157	–
Age groups		
6–9 years	36/47	0.766
10–14 years	49/74	0.662
15–18 years	24/36	0.667
Total CCDT positives	109/157	–

## Discussion

Efforts to bridge the treatment gap between children and adolescents in need of mental healthcare need to focus on supply (i.e., availability of services) and demand side factors (i.e., detection and uptake). In this study, we assessed the accuracy of a new method to overcome some of the demand side barriers by supporting community-level proactive detection of children, adolescents and families in need of mental healthcare in Sri Lanka.

### Purpose and performance of the CCDT

The CCDT is developed to proactively detect children and families in need of mental healthcare to encourage help-seeking. This may include children experiencing a mental disorder, as well as children not meeting formal diagnostic criteria but in need of mental healthcare. This study demonstrated that just over two-thirds of all children and families detected using the CCDT were correctly detected as in need of mental health treatment based on a clinical interview. This is line with the results of proactive case detection among adults in Nepal and children in Palestine and can be regarded as a moderate to high PPV since the CCDT was used among the general population [15, 19].

The CCDT vignettes focusing on children and adolescents represent generic distress domains (i.e., internalising and externalising problems) as potential indicators of mental health needs. The diagnostic criteria, which are more categorical in nature and do not include children in need of mental healthcare with subclinical levels of symptoms, were therefore used as secondary criteria. As anticipated, the predictive value of the CCDT to detect a diagnosis was lower compared to the indication for treatment. This confirms that the use of the CCDT should be limited to the detection of mental healthcare needs and not for diagnostic purposes. The performance of the CCDT is comparable to the SDQ with regards to detecting need for mental healthcare, which indicates that the CCDT could be used as a low-cost alternative to the SDQ. Even though the CCDT is not meant to detect negative cases and the sample of CCDT negatives was too small to draw conclusions, the NPV of 0.72 shows that the CCDT resulted in a relatively small percentage of false negatives.

Interpretation of these results should take the potential burden of a false positive CCDT detection in each context into account as it could cause distress among children and caregivers and may pose unnecessary pressure on a service system. Although similar burden is expected with alternative methods, proactive case detection using the CCDT is only recommended and applicable in places where services of sufficient quality are available and accessible. Furthermore, to reduce the potential burden on children and caregivers detected, it is recommended to integrate the CCDT into an existing system (e.g., training teachers in schools or community health workers engaged in home visits), and to connect caregivers with free of charge services at a convenient location.

### Constructs and performance of the CCDT

The best performing vignette, in terms of accuracy and the most cases detected, was the family vignette. The close bonds and cohesiveness in Tamil nuclear and extended families [9] may have facilitated the accurate detection of family-related problems compared to symptoms of internalising or externalising problems among children. This finding is particularly relevant given the strong emphasis on the family unit as a central pillar of life in Sri Lanka, the impact of family functioning on mental health outcomes of individuals in the family [9, 33, 34] and the rise in reported family-related issues in Sri Lanka in the past years [35]. These findings suggest that the CCDT could play an important role in proactively identifying a broad range of family-related problems at the community level. It also highlights the need for validated more in-depth family functioning assessment tools to be used after detection and designated interventions which target the family system. The majority of instruments that are currently available were developed in high income countries. Additionally, most instruments only focus on one specific element of family functioning, like parenting or communication [33]. Using the CCDT to detect family-related problems may introduce specific sensitivities compared to the detection of child mental healthcare needs. The safety of gatekeepers and the potential risks for individual family members should therefore be prioritized in any future implementation and training.

The accuracy of cases detected with the internalising vignette was slightly better compared to the externalising cases. This could be explained by cultural norms with regards to social behaviour and self-presentation. While public behaviours of self-control, obedience, and emotional restraint are important traits in Sri Lanka, behaviours that are more in line with the construct covered by the externalizing vignette such as overt extensive expression of emotions and children directly confronting an older person, are more often discouraged [36]. The differences between detecting internalising and externalising problems seem to be in conflict with dominant conceptualisations in other contexts (e.g., from Europe or North America). Here, externalising problems are often perceived as being easier to observe by an outsider and are therefore more likely to be detected and receive treatment compared to internalising problems [37]. This externalising problem vignette performed slightly better than version evaluated in Nepal [15].

### Gatekeepers and performance of the CCDT

The proactive approach relies on informal observations from individuals with strong community engagement. The type of community gatekeepers that are best placed to use the CCDT is therefore dependent on each context. The role of the gatekeepers in their community is an important general selection criteria. They should be trusted and respected individuals with easy access to families and children. In our previous study in Palestine teachers and staff working at community centres were recommended. In Sri Lanka youth club leaders, community health volunteers and active women group members were recommended by community members as users of the CCDT. Only female gatekeepers were selected as they were considered to be best placed to engage with children, adolescents and families in an effective way, conforming social norms in their community. Although this was seen as most appropriate, this meant we did not select male gatekeepers. We are therefore not able to evaluate gender differences in the results. This is something that should be explored in future research.

In this study, most cases were detected by community health volunteers. They were also best placed to engage with families in a comfortable way because of their regular home visits. Similar to the findings in Nepal, active women group members slightly outperformed community health volunteers [15]. A likely explanation for this is their more informal contacts within their daily routine and familiarity with families in their village. In our previous study in Palestine, in which teachers used the CCDT, the results were slightly better (PPV = 0.769) [19]. This may be because of teachers’ relevant educational background and training, and shows the importance of the selection of suitable gatekeepers in each new context.

## Limitations

Due to the proactive use of the CCDT to detect children and families in need of mental healthcare, the main sample included CCDT positives only. The ratio of CCDT positives and negatives (*N* = 157 vs. *n* = 29) was therefore not an accurate representation of reality. The relevant and possible accuracy metrics were therefore also limited to PPV and only a limited version of the concurrent validity could be assessed. In addition, analyses were done without prevalence rates and caution should be taken when generalising the results to other settings.

We opted for using instruments that were already available in Tamil and previously used in Sri Lanka. This introduced a couple of limitations that may have influenced the results obtained. We used an older version of the MINI-KID that was based on the DSM-IV classifications instead of the newest DSM-V. Furthermore, our sample included adolescents somewhat older than the intended age group for the SDQ (i.e., 2–17 years). Although the Tamil MINI-KID and SDQ parent version have been used in previous studies in Sri Lanka, both instruments have not been validated in Sri Lanka [28, 31]. In addition, due to a lack of available instruments that assess the global family functioning in Sri Lanka we used an instrument that had never been used in Sri Lanka. The PPV of cases detected by the family vignette was only assessed against the indication of treatment criterion. Using locally validated instruments and adjusted cut-off scores would most likely have influenced the results.

Gatekeepers first asked permission to introduce a research team member and the referral tracking sheet was only completed with their permission. This self-selection might be based on caregivers’ accurate estimation that there was no need for any mental healthcare and therefore may have inflated the results. In real-world application of the tool this potential accurate self-selection will limit the unnecessary burden on the services as help-seeking will only be encouraged. Caregivers or adolescents themselves will make the ultimate decision to seek help or not.

## Conclusions

This study demonstrates that community members using the CCDT, can accurately detect two out of three children and families in need of mental healthcare. The performance of the CCDT was comparable with the SDQ. This provides further evidence of the potential of the CCDT as an alternative scalable method to universal screening to promote help-seeking for mental health problems. Furthermore, the approach and tool could optimize the use of limited number of specialized mental health professionals by improving the match between those seeking services and the availability of care.

Overcoming under-detection is only the first step in the process of seeking help. Additional strategies are needed to tackle intersecting demand side barriers to effectively encourage help-seeking behaviour. Future research will therefore focus on the development and evaluation of an additional component of the CCDT: a help-seeking encouragement strategy.

## Supplementary Information

**Additional file 1. **The Community Case Detection Tool – English Version developed for Sri Lanka.

## Data Availability

The datasets used and/or analysed during the current study are available from the corresponding author on request.

## Abbreviations

CCDT: Community case detection tool
CIDT: Community Informant Detection Tool
DSM-IV: Diagnostic and Statistical Manual of Mental Disorders
ESCO: Eastern Self-reliant Community Organisation
ICD-10: International Classification of Diseases
MINI-KID: Mini-International Neuropsychiatric Interview for Children and Adolescents
NGO: Non-governmental Organization
LMIC: Low- and middle-income countries
PPV: Positive predictive value
RA: Research assistant
RC: Research coordinator
SD: Standard deviation
SEEK PQ-R: The Safe Environment for Every Kid Parent Questionnaire-R
SDQ: Strengths and Difficulties Questionnaire
TQS: Ten Questions Screen for Childhood Disability
WCH: War Child Holland
