# Supplementary material for: Proactive detection of people in need of mental healthcare: accuracy of the community case detection tool among children, adolescents and families in Sri Lanka
Source: Child Adolesc Psychiatry Ment Health. 2021 Oct 8;15:57. doi: 10.1186/s13034-021-00405-2 (PMC8501662; doi:10.1186/s13034-021-00405-2)
Supplement: Supplementary file 1 — Additional file 1. The Community Case Detection Tool – English Version developed for Sri Lanka. [file 13034_2021_405_MOESM1_ESM.pdf]

DO YOU KNOW OR ...

WAR child

... have you heard about a boy or girl (6-18 years) who has some or many similar problems to these? For some time, Prakash has become irritable and quick to anger. He often upsets, harasses and annoys other children, and gets into (physical) fights with others. He seems unable to control his behaviour, and argues with teachers and adults constantly. Prakash no longer cares about school; he sometimes skips classes and is getting bad marks. His behaviour has put many of his old friends off, and now he is often alone, or with others who influence him badly. He increasingly breaks rules, and for some time, there are even rumours that he has been using alcohol and stealing. All this has caused him problems at home; his parents no longer know how to deal with him. Every time they try to control him, Prakash loses his temper. Prakash does not seem happy – he does risky things, and often seems to have injuries.

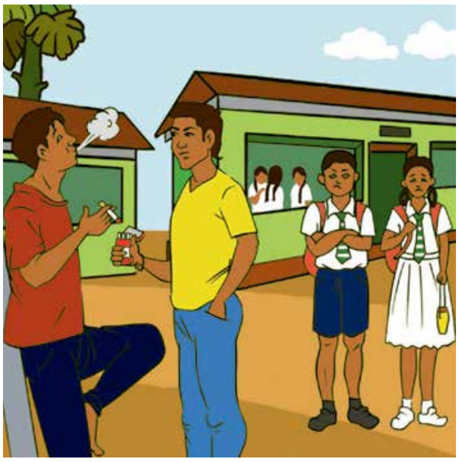
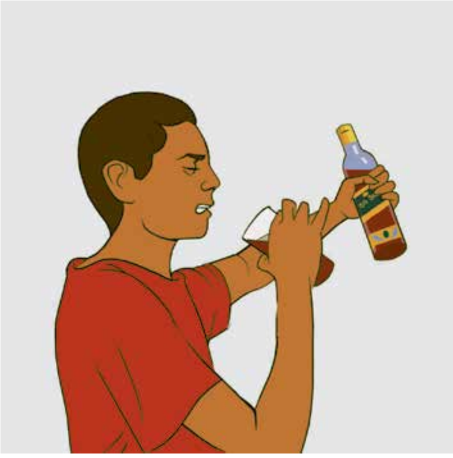
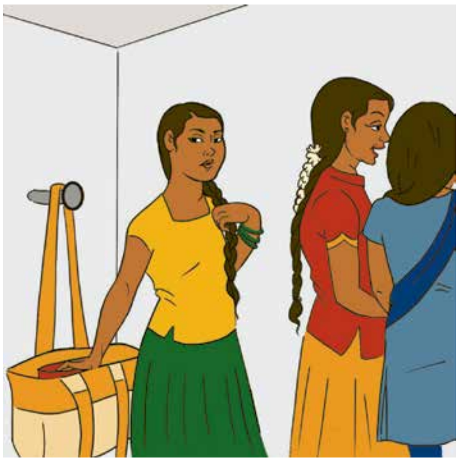
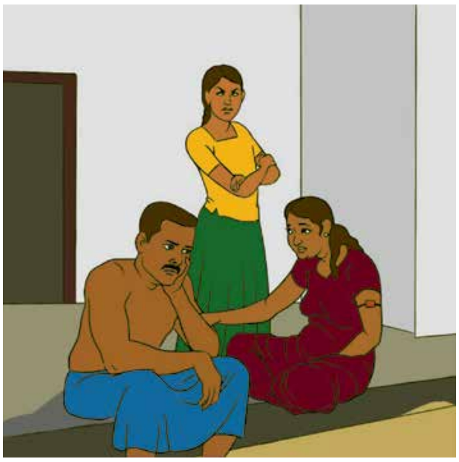
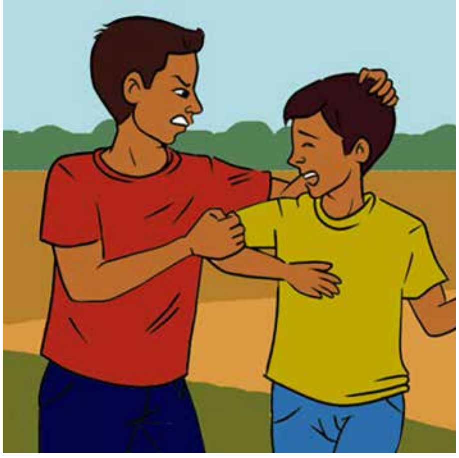
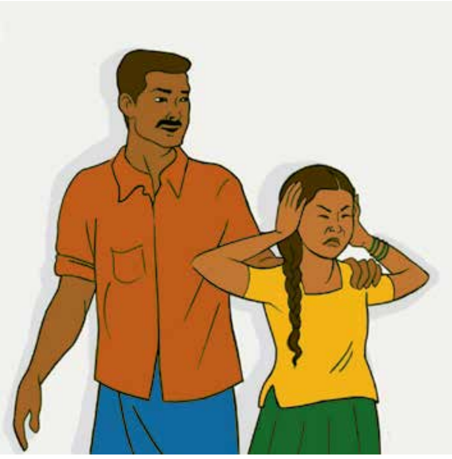

Do you know or have you heard about a boy or girl (6-18 years) who has some or many similar problems to these?

NO

YES
I know a child with some of these problems

YES
I know a child who has many of these problems

STOP

NO

Do you think these problems have been seriously affecting the child's daily life in the past month? (e.g. at school, in their family, or with friends)

YES

Invite to assessment

DO YOU KNOW OR ...

... have you heard about a boy or girl (6-18 years) who has some or many similar problems to these? Sara lives with her family and goes to school in her village. She has always enjoyed going to school and playing with her friends. But in the last few months Sara has not been acting the way she used to. She is no longer as energetic or excited to play her favourite game “Karem-board” or be with her friends, and often seems to be sad or irritable. Even eating nice food does not make her happy like it used to. Most mornings Sara complains of a headache or stomachache and wants to stay home. She remains quiet more often than before and always seems tired. It seems like Sara is always thinking and worrying too much, and cannot concentrate as well as before. Her marks have deteriorated at school and she is no longer involved in many activities – instead she often wants to sit alone. Sara does not seem to feel good about herself and when asked what she wants to become in the future, she cannot think of a positive answer.

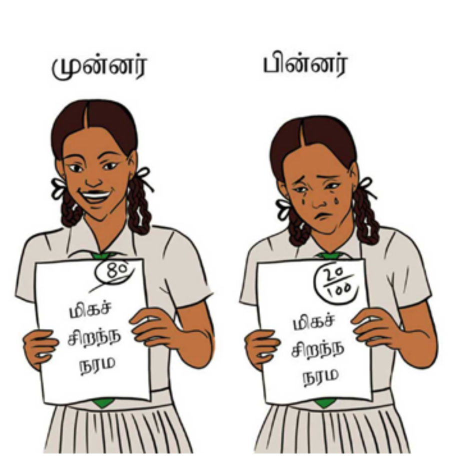

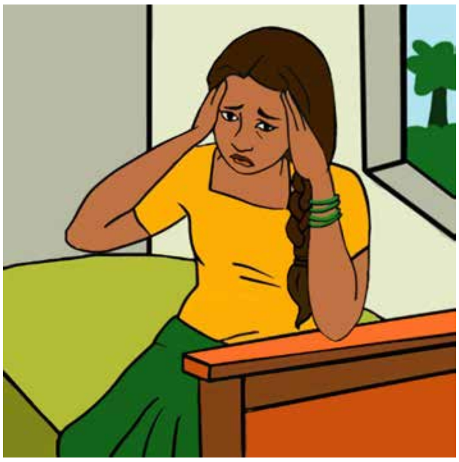

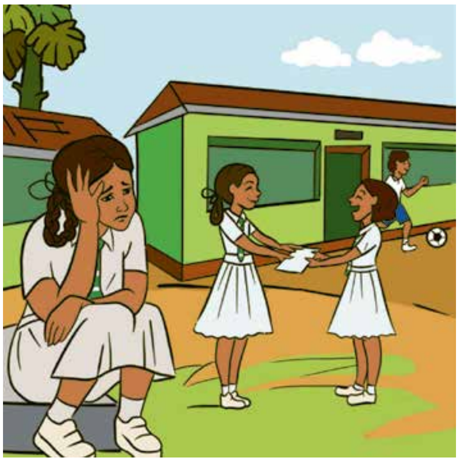

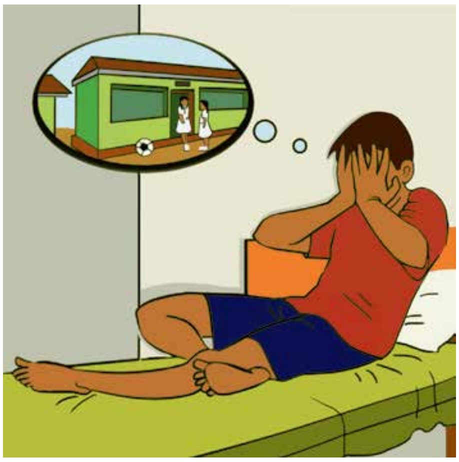

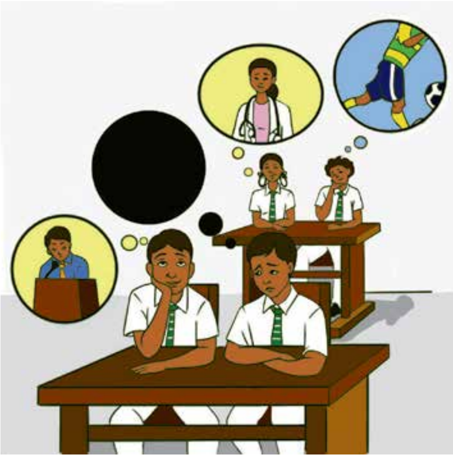

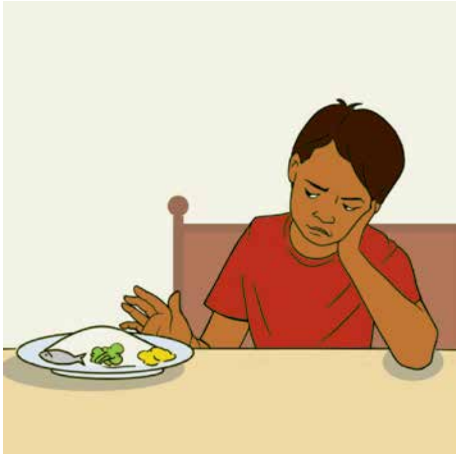

Do you know or have you heard about a boy or girl (6-18 years) who has some or many similar problems to these?

NO	YES<br>I know a child with some of these problems	YES<br>I know a child who has many of these problems
STOP	Do you think these problems have been seriously affecting the child’s daily life in the past month? (e.g. at school, in their family, or with friends)	Invite to assessment

NO → STOP

YES → Invite to assessment

DO YOU KNOW OR ...

... have you heard about a boy or girl (6-18 years) who has some or many similar problems to these? Ramani lives with her parents and four siblings. Both parents are often away from home and not able to care and protect Ramani and her siblings. Ramani and her siblings are often left to take care of themselves, and this is too difficult for them. This shows in her recent weight loss, her physical appearance; she is often dirty, disorganised, and tired compared to others in her village. One time when Ramani was seriously ill, there was no one to take her to the medical centre. For some time now, Ramani's father has started taking alcohol extensively and is not able to go to work anymore. When both parents are at home, they often argue violently in front of their children and her father even sometimes beats his wife and children. Since some time now, the people in the village haven't seen Ramani's mother at the mother society meetings where she used to go.

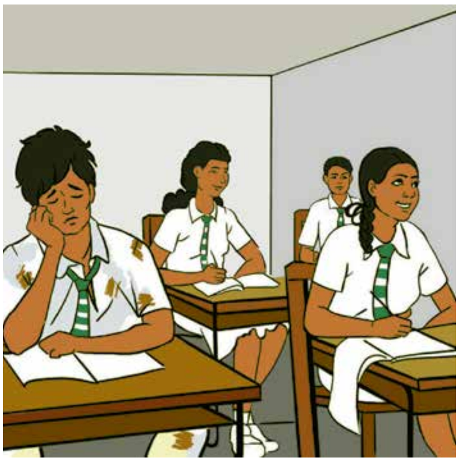
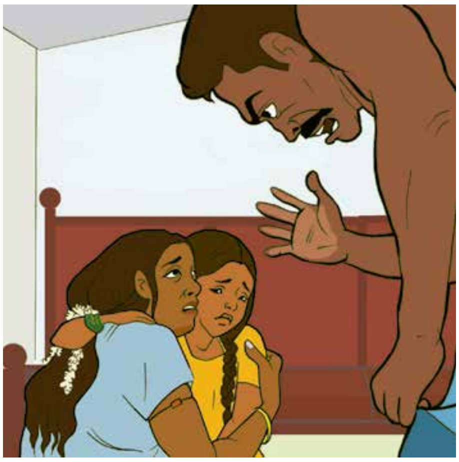
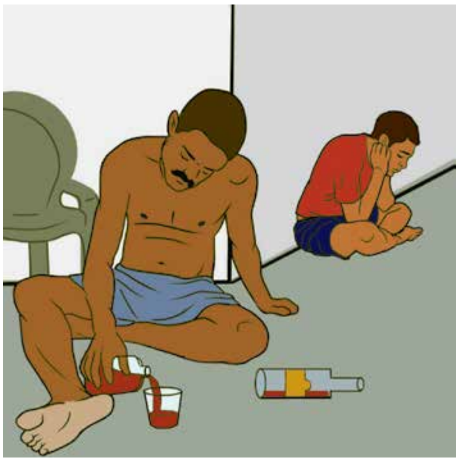
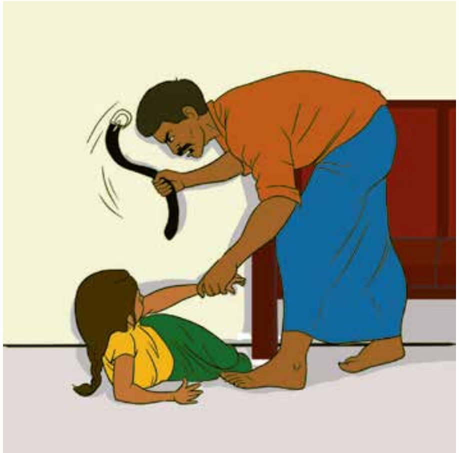
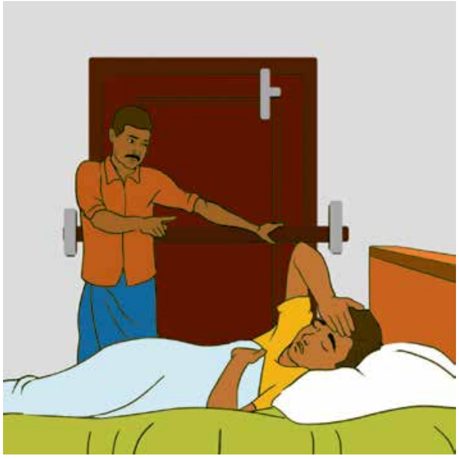
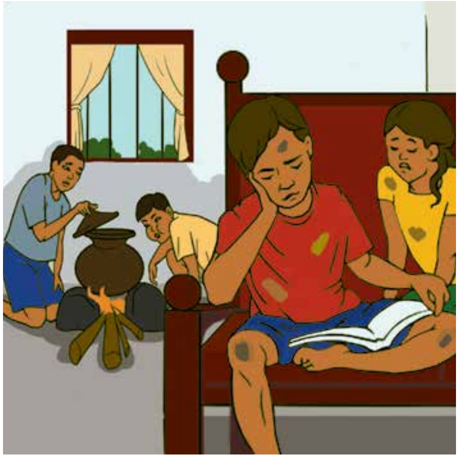

Do you know or have you heard about a boy or girl (6-18 years) who has some or many similar problems to these?

- **NO** → **STOP**
- **YES – I know a child with some of these problems** → **Do you think these problems have been seriously affecting the child's daily life in the past month? (e.g. at school, in their family, or with friends)**
  - **NO** → **STOP**
  - **YES** → **Invite to assessment**
- **YES – I know a child who has many of these problems** → **Invite to assessment**
